# Rewind for Posttraumatic Stress Disorder: A Randomised Controlled Trial

**DOI:** 10.1155/2023/6279649

**Published:** 2023-10-12

**Authors:** Laurence Astill Wright, Kali Barawi, Neil Kitchiner, Danielle Kitney, Catrin Lewis, Alice Roberts, Neil P. Roberts, Natalie Simon, Cono Ariti, Ian Nussey, David Muss, Jonathan I. Bisson

**Affiliations:** ^1^Division of Psychological Medicine and Clinical Neurosciences, Cardiff University School of Medicine, UK; ^2^Centre for Academic Mental Health, Population Health Sciences, University of Bristol, UK; ^3^Directorate of Psychology and Psychological Therapies, Cardiff & Vale University Health Board, Cardiff, UK; ^4^Centre for Trials Research, Cardiff University, UK; ^5^School of Engineering, Cardiff University, UK; ^6^International Association for Rewind Trauma Therapy, UK

## Abstract

**Background:**

The Rewind Technique (Rewind) is a psychological therapy for people with posttraumatic stress disorder (PTSD), which is already used regularly in the National Health Service (NHS), the third sector and private practice across the UK. This study set out to explore the potential efficacy and feasibility of remotely delivered Rewind for the treatment of PTSD.

**Methods:**

This study was a two-armed, exploratory RCT to assess the preliminary efficacy, adherence, feasibility, and factors affecting outcome of Rewind versus a waitlist control group.

**Results:**

The entire trial was delivered remotely using video calls for treatment sessions and outcome assessments. A total of 40 participants were randomised with 80% retention at the primary endpoint of 8-week postrandomisation. The difference in Clinician-Administered PTSD Scale for DSM-5 scores between the immediate and delayed Rewind arms was 12.64 (95% CI, 2.29 to 22.99, *F* = 6.38, df = 1, *p* = 0.02) at 8 weeks. The Cohen's *d* was 1.05 indicating a large effect size at 8 weeks with maintenance in symptom improvement at 16 weeks.

**Conclusion:**

Rewind, delivered remotely, demonstrated a large effect size in treating symptoms of PTSD within this trial cohort. This trial demonstrates a preliminary signal of efficacy supporting the clinical use of Rewind in the treatment of people with PTSD.

## 1. Introduction

The Rewind Technique (Rewind) [1] is a psychological therapy used in the treatment of people with symptoms of posttraumatic stress disorder (PTSD). Despite being used regularly in the National Health Service (NHS), the third sector and private practice (IARTT) across the UK, there is an absence of robust evidence to support its routine delivery, although results from nonrandomised trials [2, 3] have been encouraging. Rewind employs a “rewinding” technique—where the participant imagines that they are in a cinema watching a film of her/his traumatic event, the participant then enters the screen and relives their trauma memory in reverse very quickly. It is theorised that this rewinding may utilise mechanisms such as exposure, extinction, and reconsolidation to alleviate PTSD symptoms [4]. Other therapies based on Rewind, e.g., the Reconsolidation of Traumatic Memory (RTM) protocol, have demonstrated high effect sizes in the treatment of PTSD [5]. A recent systematic review however noted low confidence in the effect size estimate and the trials being at high risk of bias [6].

While the precise mechanism of action of Rewind has not yet been fully elucidated, the therapy is aimed at briefly activating a traumatic memory and then eliciting dissociative experiences surrounding the trauma which are proposed to facilitate reconsolidation of the memory, by decreasing the emotional salience the memory evokes and thus decreasing PTSD symptomatology [7]. The brief memory mobilisation stimulus is thought to be too brief to produce effects via improving the extinction of the traumatic memory [8, 9]. Follow-up suggests that the rapid reacquisition or reinstatement of traumatic memories that might be expected if the underlying mechanism was one of memory extinction does not occur [5]. Reconsolidation has thus been proposed as a possible mechanism of action, rather than memory extinction [6, 10, 11].

While trauma-focused psychotherapies (specifically cognitive behavioural therapy with a trauma focus and eye movement desensitization and reprocessing) have medium to large effect sizes [12, 13], these therapies require large amounts of therapist time, and thus, treatment can be inaccessible for many individuals who are likely to benefit from it [13], with lengthy waiting lists in many clinical services. Promising evidence from nonrandomised trials [2, 3] suggests that Rewind could have the potential to have a similar effect size to existing trauma-focused psychotherapies, but be more time- and cost-efficient, delivering a trauma-focused intervention in up to three 60-minute sessions. Recent research has demonstrated that less intensive interventions may be comparably effective [14]. As Rewind is relatively simple to deliver (full intervention protocol detailed in methods), it also has the potential to be delivered by therapists who require less formal training than for currently recommended treatments and might, therefore, be more easily scalable. Furthermore, evidence of multiple effective therapies would provide more treatment choices for people with PTSD, and some individuals may prefer Rewind as it does not require detailed trauma disclosure.

We undertook a preliminary efficacy randomised controlled trial (RCT) to determine if Rewind is likely to be a good candidate for the treatment of PTSD, as per our protocol [4]. Our objectives were as follows:

1. To investigate the effect size of Rewind at reducing PTSD symptoms in people with PTSD
2. To establish whether any symptom improvement was maintained over 16-week follow-up
3. To investigate the impact of the Rewind Technique on symptoms of depression, anxiety, and insomnia
4. To investigate if an effectiveness of RCT is feasible and indicated

## 2. Material and Methods

### 2.1. Design

This study was a two-armed, exploratory RCT to assess the preliminary efficacy, adherence, feasibility, and factors affecting outcome of Rewind versus a waitlist control group. Due to COVID-19 restrictions, the entire trial was delivered remotely using video calls for treatment sessions and outcome assessments.

### 2.2. Sample Size

As per our protocol [6], we conservatively based the power calculation for this study on the broad range of effect sizes reported for trauma-focused psychological therapies for PTSD and considered an effect size of 1.5 highly clinically relevant [13, 15]. To detect an effect size of 1.5 with 80% power and a 5% significance level, 15 participants were required in each arm. Allowing for a 25% attrition rate [13], a total proposed sample size of 40 was determined.

### 2.3. Inclusion/Exclusion Criteria

We adopted a pragmatic approach and employed broad eligibility criteria, as our interest was in determining the efficacy of Rewind for people with PTSD presenting to the UK's National Health Service (NHS).

Inclusion criteria were as follows: adults aged 18 or over, able to provide informed consent, English language fluency, and met DSM-5 criteria for PTSD secondary to a single traumatic event [6].

Exclusion criteria were as follows: complex PTSD, current psychosis or bipolar disorder, traumatic brain injury, substance dependence, acute suicidal ideation, learning disability, previous or current receipt of an adequate trial of trauma-focused psychological treatment for PTSD, change to the type or dosage of psychotropic medication within one month of baseline assessment, and insufficient IT to engage with online trial. Comorbidity with other mental disorder was permitted if PTSD was the primary condition. Pretreatment comorbidity was assessed using clinical presentation, past psychiatric history, and self-report [6].

### 2.4. Recruitment and Consent

Ethical approval was granted by Wales Research Ethics Committee 2 in March 2020. Trial registration was ISRCTN91345822. Potentially eligible study participants attending primary and secondary care NHS mental health clinics [16] were approached by clinicians involved in their care and told about the study. They were then screened according to the eligibility criteria and then fully assessed by a member of the research team. Recruitment spanned from March 2020 to November 2021.

Participants who were eligible for inclusion were asked to monitor their symptoms for two weeks using a daily self-report diary as symptom monitoring alone has been found to reduce PTSD symptoms and cause the loss of diagnostic status for some people [17]. Following this, participants provided written informed consent. The study team then collected baseline demographic data and outcome measurements, reassessed eligibility (prior to randomisation), and then randomised eligible individuals in randomised blocks of four and six in a 1 : 1 ratio, using an online randomisation application [18]. One group received Rewind immediately; the other was allocated to a waitlist for eight weeks prior to then receiving the intervention.

### 2.5. Outcome Measures

A trained member of the research team, blinded to randomisation, conducted all clinical outcome assessments. Because of the nature of the intervention, it was not possible for therapists or participants to be blinded to treatment allocation, but participants were asked not to discuss their allocation with their assessor.

The primary outcome of PTSD symptom severity at 8-week postrandomisation and PTSD diagnosis was measured using the Clinician-Administered PTSD Scale for DSM-5 (CAPS-5) [19] administered by 5 trained postgraduate researchers. The outcome raters demonstrated moderate interrater reliability based on training videos with a kappa of 0.60. The CAPS-5 is widely considered the gold standard in DSM-5 PTSD assessment, demonstrating high internal consistency (*α* = .88) and strong test-retest reliability (*к* = .83) [19]. PTSD symptom severity and PTSD diagnosis at 16-week postrandomisation were also measured using the CAPS-5.

Secondary self-reported outcomes, collected at 8 and 16 weeks, were the PTSD Checklist (PCL-5), a validated self-report measure for DSM-5 PTSD symptoms [20, 21]; the International Trauma Questionnaire (ITQ), a validated and widely used self-report measure for ICD-11 PTSD and complex PTSD [22]; the Patient Health Questionnaire-9 (PHQ-9), a validated self-report measure for the assessment of DSM-5 depressive symptoms [23]; the Generalised Anxiety Disorder Assessment-7 (GAD-7), a self-report measure for assessing symptoms of generalised anxiety disorder [24]; the Insomnia Severity Index (ISI), a validated self-report measure for symptoms of insomnia over the past month [25]; and the five-level EQ-5D [26], a validated self-report measure for health-related quality of life [26]. We monitored changes in PTSD using the PTSD Checklist (PCL-5) at the start of each treatment session, in addition to the 8- and 16-week assessments. Dropout numbers gave an indication of feasibility [4]. Fidelity of treatment according to the Rewind protocol was assessed, and therapists were asked to audio record at least one session. Recordings were then rated by the intervention developer DM using a fidelity checklist specifically developed for the trial.

### 2.6. Intervention

#### 2.6.1. Rewind

Up to three 60-minute sessions were delivered following a protocol developed by one of the coauthors, DM [1], and modified by the research team following feedback from psychological therapists delivering the intervention before the trial began. The intervention was administered by experienced and trained psychological therapists within the Cardiff and Vale University Health Board Traumatic Stress Service and Veterans NHS Wales under the supervision of DM. The therapists were trained in the Rewind Technique over two and a half days and were required to satisfactorily treat two people with PTSD with Rewind before treating trial participants. Group supervision with DM occurred for one hour fortnightly with DM available for consultation between supervision sessions. After being introduced to the technique, the participant was asked to imagine he/she was in a cinema watching a film of her/his traumatic event as if it had been captured on CCTV. Rather than the film start at the trauma itself, the participant was told the film starts just before the traumatic event took place, when all was well. This was then followed by the targeted memory which included all the images, sounds, smells, and other sensory features plus (if this was part of the regular recall) what the participant feared could have happened next but did not. Once the recall ended, if the traumatic event was directly experienced (as opposed to witnessed), the participant was (metaphorically) invited to enter the screen and, at that point, the film was rewound at speed back to the exact starting point (where all was well before the trauma). The aim was for the forward part of the process of recalling the trauma to last up to approximately 2 minutes and the rewind part about 10 seconds. The participant was usually required to practice the technique a few times to make sure the participant was following all of the components of the intervention correctly [4]. At the second and third sessions, the Rewind was repeated and refined if the participant continued to describe significant symptoms. If the participant no longer reported any distressing symptoms, any subsequent sessions were cancelled.

#### 2.6.2. Wait List

No intervention was received for 8 weeks after randomisation, following which the participants then received Rewind in the same manner as the immediate treatment group.

### 2.7. Analyses

For quantitative outcome data, the means of continuous outcome data were compared using ANCOVA, with the baseline CAPS-5, baseline PHQ-9, gender, and duration of PTSD symptoms as covariates, as per our a priori agreed statistical analysis plan. Analyses were undertaken on an intention to treat basis using a complete case analysis. Sensitivity analyses at 8 weeks were undertaken using multiple imputations. The CAPS-5 at 8 weeks was imputed using the chained equation approach of van Buuren. Twenty imputed data sets were produced and the parameter estimates combined using Rubin's rules to estimate the effect of the intervention [27]. The analyses were performed at the end of data collection using SPSS version 27 ([28], IBM SPSS Statistics for Windows) and Stata version 17 [29].

## 3. Results

### 3.1. Recruitment and Retention

**Figure 1:**
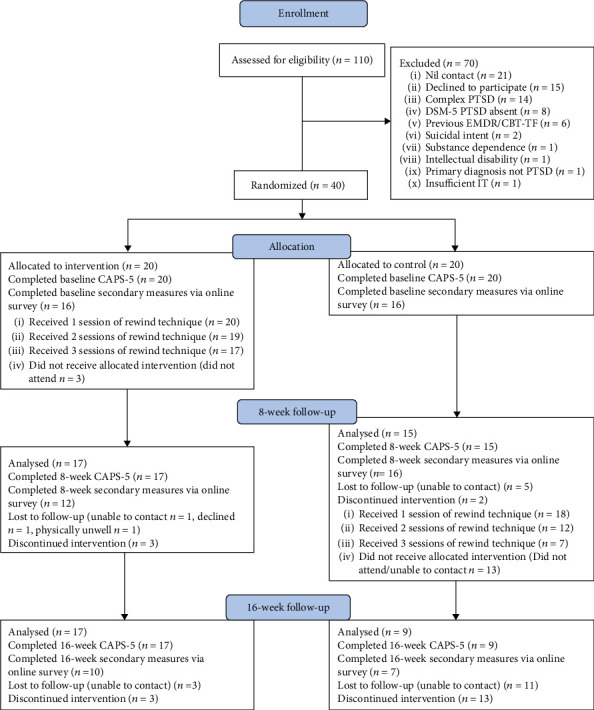
CONSORT 2010 flow diagram.

110 people with suspected PTSD were referred to the trial with 40 participants randomised (CONSORT flowchart, Figure 1).

### 3.2. Background Information

Table 1 summarises participant demographics, which were similar in both the intervention and control groups.

Table 2 documents the primary traumatic events reported by participants and categorised using the LEC—the most common being sexual assault and life-threatening illness or injury.

Supplementary Table 1 is the CONSORT RCT checklist.

### 3.3. Outcome Data

**Figure 2:**
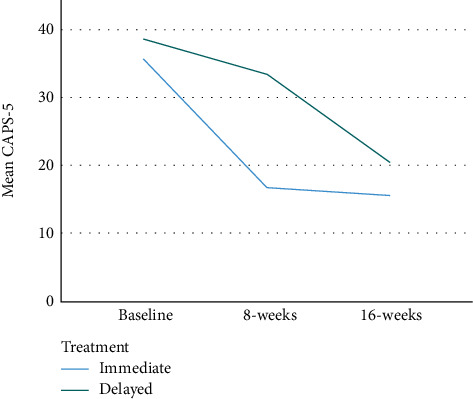
Mean CAPS-5 over time by randomisation arm without imputation.

Table 3 and Figure 2 document data on outcome measures at baseline and 8 and 16 weeks, while Table 4 documents primary and secondary outcome analyses for differences between 8 and 16 weeks. 35 participants were analysed at 8 weeks (primary outcome) with 5 in the delayed treatment group lost to follow-up. The difference in CAPS-5 between the immediate and delayed arms was 12.64 at 8 weeks (95% CI 2.29 to 22.99, *p* = 0.019) and 4.25 (95% CI -9.50 to 18.00, *p* = 0.525) at 16 weeks. The between-subject Cohen's *d* was 1.05 and the partial eta squared was 0.217, indicating a large effect size at 8 weeks with maintenance in symptom improvement at 16 weeks. There were no statistically significant differences between the groups on the AUDIT-O MSPSS and ISI.

All 40 participants met the DSM-5 criteria for a diagnosis of PTSD on the CAPS-5 at baseline. Of the 20 who received immediate Rewind, 10 participants at week 8 no longer met the criteria in the intervention group, with 7 remaining PTSD positive (the remaining 3 participants dropped out). In the delayed treatment group, 3 participants at week 8 no longer met the CAPS-5 criteria, with 12 remaining PTSD positive (the remaining 5 participants dropped out). Once all the participants had been offered intervention at 16 weeks, 18 participants were CAPS-5 negative, with 8 remaining positive (*n* = 26) (Supplementary Table 2). No adverse effects were noted during the study.

The 40 participants attended a mean of 2.35 sessions (SD: 0.92). 24 participants attended all 3 sessions, 8 participants attended 2 sessions, 6 participants attended 1 session, and 2 participants did not attend any. Three of these participants withdrew from the study, while the others had sufficient symptom amelioration to not attend subsequent sessions via self-report and therapist agreement. The mean number of rewind loops received across all attended sessions for 38 participants was 6.18 (SD: 2.76).

Analyses were undertaken on an intention-to-treat basis. Sensitivity analyses at 8 weeks were undertaken via multiple imputations—there was little change to the primary or secondary outcomes with the imputation of missing data at 8 weeks.

### 3.4. Fidelity

Audio recordings of 13 treatment sessions were rated for fidelity to Rewind for 11 different participants. Overall, fidelity was rated as high. One session was rated as inadequate, three sessions were rated as fair, one session was rated as good, and eight sessions were rated as very good.

## 4. Discussion

Rewind was an efficacious treatment for people with PTSD in this feasibility RCT delivered to protocol with high fidelity. The mean difference in CAPS-5 score of 12.64 between the two arms of the trial at 8 weeks is likely to be highly clinically significant [30]. Those on the waitlist experienced a mean drop of 4.85 on the CAPS-5 at 8 weeks, possibly reflecting some sort of ameliorative expectancy effect. A similar pattern of improvement was observed on some, but not all, of the secondary outcome measures. For example, AUDIT scores demonstrated low-risk drinking at baseline, and thus, we may not expect to see an improvement here. These findings are reflected in some other PTSD trials [31] but contrast to other waitlist controlled trials where participants do not improve and sometimes deteriorate, possibly expecting change only after receiving the intervention [32].

The total reduction of 16.94 points in CAPS-5 scores from pre- to post-Rewind in those receiving the therapy immediately is a 48.4% reduction. The reduction in symptoms was maintained in the immediate treatment group at 16-week follow-up. The PTSD symptom severity reduction demonstrated here also appears to be related to major improvements in functioning and quality of life, as demonstrated by secondary outcome measures, e.g., the WSAS. The effect size of Rewind demonstrated in this trial (Cohen's *d*: 1.05), while clinically significant, is smaller than other Rewind [2, 3] (Cohen's *d*: 1.58–1.71) and RTM studies ([5, 7]) (SMD = 3.64 [4]), where methodological flaws (e.g., nonrandomised, nonblinded outcome assessments) may have exacerbated treatment effects [6]. It is also possible that RTM is a more effective therapy than Rewind, or sample/inclusion criteria differences led to different effects. We believe that our trial overcomes the methodological issues of previous work and thus provides greater certainty in the degree of effect size. The effect size is comparable to other trials of trauma-focused psychological interventions with the waitlist control groups [13]. Similar to previous studies [2, 3], no adverse effects were noted.

### 4.1. Mechanism of Action

This trial was not designed to determine a potential mechanism of action, and the purported mechanism of reconsolidation [4] remains speculative as dismantling studies are required to determine this. Reconsolidation mechanisms may be present, and, as noted above, the brief memory mobilisation stimulus utilised by Rewind seems likely to be too short to produce effects via extinction, which typically requires longer periods of memory mobilisation [9]. This requires clarification, and a combination of therapeutic mechanisms may be possible. The therapy, for example, employs third-person techniques to decrease avoidance, and the emotional salience of focusing on the trauma memory should be considered.

### 4.2. Strengths and Limitations

This is the first randomised controlled trial to evaluate Rewind, with previous nonrandomised trials [2, 3] demonstrating promise but with a low quality of evidence. This trial gives a preliminary signal of efficacy, supporting the clinical use of Rewind in the treatment of people with PTSD. This was a rigorous, remotely delivered RCT, adhering to a prepublished protocol and to CONSORT guidelines [33]. A strength of the trial was the careful supervision and training of the psychological therapists, with concomitant fidelity checks on 13 treatment sessions demonstrating good adherence to the Rewind treatment protocol, although the other treatment sessions were not assessed. Some therapists, however, reported gaining confidence as they became more familiar with the technique, and it may be that earlier participants could have done better had the therapists at that point had more experience and confidence with the technique.

While the attrition rate (20%) was lower than expected for the primary outcome—8-week CAPS-5—there was only attrition in the delayed treatment group suggesting the possibility of some bias as a result of this differential (Supplementary Table 3). The attrition was much higher for the 16-week assessments, in particular for the control group, as we were unable to collect data on the participants who were lost to follow-up. The imputation of missing data at 8 weeks showed little difference in primary and secondary outcomes. Due to the paucity of the data at 16 weeks, it was not possible to conduct an imputation. This raised the possibility for attrition bias at 16 weeks. This control group attrition may demonstrate that being allocated to a waitlist is disheartening and may lead to comparatively higher dropout than the immediate treatment arm. In the delayed treatment group, there was considerable dropout between sessions 1 and 3 of Rewind (*n* = 11, 55%) raising some questions such as the acceptability of the therapy to these participants, although there may also be other reasons for this, e.g., symptom improvement after 1 or 2 sessions as suggested by previous observational studies [2, 3].

The control group further had slightly higher baseline CAPS-5 scores (3.65 difference), and this greater PTSD severity may have contributed to a higher attrition rate [13, 34]. This, however, is still a small difference and an expected result of randomisation. While using pragmatic inclusion/exclusion criteria was a key strength of this trial, it may also have led to higher attrition rates. Attrition was, however, generally comparable to that of other trials of trauma-focused psychological therapy for people with PTSD, trials which are associated with higher avoidance and dropout [30]. In addition, the interrater reliability was only good/moderate at 0.60.

The trial was relatively small, with a sample size of 40, and this must be acknowledged when drawing inferences from the results. Furthermore, the follow-up period was restricted to 4-month postrandomisation, so we cannot be certain about the longevity of treatment effects beyond the primary outcome at 8 weeks. It is impossible to adequately conduct double-blind trials of psychological interventions, but the inclusion of a waitlist serves as an appropriate control for this study. There is, however, consequently a risk of performance bias, as participants and therapists could not be blinded to the fact that individuals in the immediate treatment group were receiving Rewind.

Interpretation is made more challenging by the improvement of participants in the waitlist control before they received Rewind and highlights the challenges in identifying a “perfect” control condition. The improvement in the waitlist group could be attributed to expectation/regression to the mean effects, e.g., participant help seeking at perceived symptom nadir. Despite control group improvement, there was still statistically and clinically significant improvement in the intervention group, further adding weight to the positive findings as greater than any possible regression to the mean effect, for example. The cross-over design also provided further evidence of the longevity of effects, demonstrating the further improvement post-Rewind in the immediate treatment group. The reduction in PTSD symptomatology from Rewind was significantly larger than just an expectation effect, but nonetheless, psychological therapies typically produce and harness placebo effects, and this may have contributed to the reported effect size, although this is a limitation of psychological treatment trials more broadly [35]. The trial results may also have been influenced by the entire trial being conducted during the COVID-19 pandemic. For example, some research indicates those with PTSD may have experienced worsened mental health during the pandemic, which may have influenced outcomes related to the trial [36].

### 4.3. Clinical and Research Implications

As this trial is preliminary to an equivalence trial comparing Rewind Technique to more widely used trauma-focused therapies, such as CBT-TF or EMDR, it would be premature to recommend it for routine clinical practice. This work, however, suggests that the current use of Rewind as a therapy in the UK does not worsen the symptoms of people with PTSD, although CBT-TF remains the recommended psychological intervention with the best evidence base [13]. It is important that only evidence-based interventions are routinely delivered to people with PTSD, and this trial makes significant improvements in establishing the evidence base for Rewind by demonstrating efficacy in this setting.

While CBT-TF and EMDR are efficacious therapies with large effect sizes [13], they require many more hours of therapist time than Rewind, with some trauma-focused therapies requiring 12-20 sessions [30]. One to three sessions of Rewind demonstrate potential as a more time- and cost-efficient trauma-focused intervention than CBT-TF and EMDR but with a similar effect size. It could also provide an additional treatment option for people with PTSD. There is a need, however, for careful RCT evaluation against CBT-TF and EMDR to determine the relative effectiveness of Rewind compared to these established treatments. If Rewind is at least as effective as CBT-TF and EMDR, it could play a major role in increasing the availability of evidence-based treatments for PTSD. Rewind has very strong potential scalability—it is relatively simple to deliver and, although not tested in this trial, thus has the potential to be delivered by low-intensity psychological therapists. This could potentially be delivered within services where current access to trauma-focused interventions is extremely limited and marred by long waitlists. The further development of Rewind in larger trials may also align well with key ambitions to increase digitally enabled therapies in future models of service delivery [37, 38].

The entire trial was delivered remotely using telephone/video calls for treatment sessions and outcome assessments, and the positive findings within this setting have further clinical and research implications of this treatment, e.g., scalability and flexibility of both future trials and of the treatment itself for people with PTSD. It is possible that the remote delivery of Rewind in this trial may have impacted the observed effects as Rewind is normally delivered face-to-face. Rewind's efficacy through remote delivery adds to existing literature supporting the remote delivery of psychological therapy to people with PTSD [39] and thus potential scalability. Further research is required although many trials are currently in progress [40]. As the selection processes in this trial demonstrate, however, many people remain unable or do not wish to engage with digital approaches [41]. Barriers encountered to consistently accessing technology for some participants and connectivity issues may have further influenced participants' ability and willingness to attend further sessions and assessments, possibly also influencing dropout.

Remote Rewind may represent a promising low-cost therapy with minimal barriers to delivery as a first-line intervention, as part of a stratified care approach. While many individuals improved dramatically following Rewind, some participants did not. It is therefore possible that a personalised approach may be more efficacious, decreasing or increasing the number of sessions as indicated, similar to a dose response effect. Furthermore, the high attrition rate in the control group between sessions 1 and 3 of Rewind (*n* = 11, 55%) suggests potential scope for improvement via personalised adaptation of the psychological therapy [42].

The suggested alterations should be further evaluated in implementation work as part of future research whilst also testing Rewind against existing trauma-focused therapies for definitive effectiveness, alongside cost-effectiveness work to allow future informed decisions to be made around clinical commissioning and adoption within existing services. This would also allow an alternative intervention-based control group compared to the waitlist control evaluated here. As noted above, the actual mechanism Rewind utilises to achieve an amelioration of PTSD symptoms is unclear and may involve exposure and/or reconsolidation mechanisms [6]. The mechanism could be further explored/elucidated with back translational work.

## Figures and Tables

**Table 1:** Demographics and clinical characteristics.

	Immediate treatment arm, *n* (%), mean|SD	Delayed treatment arm, *n* (%), mean|SD	Total, *n* (%), mean|SD
*n*	20	20	40
Age	38.16 | 12.75	36.46 | 13.44	37.31 | 12.96
Female gender	9 (45)	14 (70)	23 (57.5)
Ethnic origin			
White Welsh/English/Scottish/Northern Irish/British/Irish/Gypsy or Irish Traveller/Roma/any other White background	19 (95)	18 (90)	37 (92.5)
Mixed or multiple ethnic background	0 (0)	1 (5)	1 (2.5)
Asian/Asian British/Black/Black British/Caribbean/African/Arab/any other ethnic group	1 (5)	0 (0)	1 (2.5)
Highest level of qualification			
Degree level or above	8 (40)	3 (15)	11 (27.5)
2+ A levels or equivalent	2 (10)	7 (35)	9 (22.5)
5+ GCSEs or equivalent	2 (10)	5 (25)	7 (17.5)
1-4 GCSEs or equivalent	4 (20)	2 (10)	6 (15.0)
Apprenticeship	1 (5)	1 (5)	2 (5.0)
Other qualifications	2 (10)	1 (5)	3 (7.5)
No qualifications	1 (5)	0 (0)	1 (2.5)
Current employment status			
Employed	14 (70)	11 (55)	25 (62.5)
Student	2 (10)	6 (30)	8 (20.0)
Retired	1 (5)	1 (5)	2 (5.0)
Unable to work	2 (10)	1 (5)	3 (7.5)
Unemployed and looking for work	1 (5)	0 (0)	1 (2.5)
Possible major depressive disorder (PHQ ≥ 10)			
Yes	16 (80)	17 (85)	33 (82.5)
No	4 (20)	2 (10)	6 (15)
Duration of PTSD symptoms in months (not time since trauma)	42.83 | 70.32	30.38 | 37.22	36.60 | 55.89
Directly experienced index trauma	12 (60)	12 (60)	24 (60)
Witnessed index trauma	8 (40)	8 (40)	16 (40)
Index trauma included sexual violence	3 (15)	4 (20)	7 (17.5)

**Table 2:** Primary traumatic event.

Primary traumatic event	*N* (%)
Sexual assault	6 (15)
Life-threatening illness or injury	6 (15)
Sudden accidental death	5 (12.5)
Serious accident at work, home, or during recreational activity	4 (10)
Physical assault	3 (7.5)
Assault with a weapon	3 (7.5)
Serious injury, harm, or death you caused to someone else	3 (7.5)
Transportation accident	2 (5)
Combat or exposure to a warzone	2 (5)
Severe human suffering	2 (5)
Any other very stressful event or experience	2 (5)
Sudden violent death	1 (2.5)
Childhood physical abuse	1 (2.5)

**Table 3:** Primary and secondary outcome results at 8- and 16-week follow-up for nonimputed data.

		Immediate treatment group			Delayed treatment group		
		*n*	Mean	SD	*n*	Mean	SD
CAPS-5 symptom severity	Baseline	20	36.50	8.24	20	40.15	5.77
	8 weeks	16	18.06	14.96	15	35.00	9.20
	16 weeks	17	16.94	14.51	9	21.33	12.99
PCL-5	Baseline	20	52.65	11.56	19	57.21	12.31
	8 weeks	12	21.25	20.41	16	44.13	14.83
	16 weeks	10	24.50	24.29	7	23.71	21.68
WSAS	Baseline	19	21.16	9.44	18	23.39	9.85
	8 weeks	11	10.27	10.78	16	22.88	9.24
	16 weeks	9	14.67	13.36	6	13.50	13.82
PHQ-9	Baseline	20	14.65	6.17	19	17.74	5.65
	8 weeks	12	5.58	6.72	16	15.06	6.14
	16 weeks	10	7.70	8.56	7	8.29	7.11
GAD-7	Baseline	20	13.10	4.73	19	14.53	4.27
	8 weeks	12	5.75	6.20	16	12.69	4.85
	16 weeks	10	7.20	6.78	7	5.43	4.69
ISI	Baseline	20	17.65	5.96	19	19.00	6.21
	8 weeks	12	10.00	8.42	16	17.06	6.81
	16 weeks	10	11.00	10.85	7	12.57	5.32
Audit-O	Baseline	18	5.61	8.64	17	4.29	5.62
	8 weeks	11	4.36	6.12	16	4.25	4.84
	16 weeks	10	3.40	5.42	7	3.86	4.74
MSPSS	Baseline	20	56.75	15.95	19	54.89	15.81
	8 weeks	12	63.25	14.72	15	52.80	17.80
	16 weeks	10	64.00	16.81	7	54.43	22.10

**Table 4:** Primary and secondary outcome analyses for differences between 8 and 16 weeks using completer only data.

		Time point										
		Immediate				Delayed				Difference in means	95% confidence interval	*p* value
		*n*	Mean	SD	95% confidence interval	*n*	Mean	SD	95% confidence interval			
CAPS-5	Baseline	20	37.79	1.66	34.41 to 41.17	20	39.69	1.66	36.302 to 43.067	1.896	-3.110 to 6.901	0.446
	8 weeks	16	20.25	3.34	13.33 to 27.16	15	32.88	3.47	25.71 to 40.05	12.64	2.287 to 22.986	**0.019**
	16 weeks	17	18.00	3.63	10.37 to 25.63	9	22.25	5.24	11.24 to 33.26	4.25	-9.50 to 18.00	0.525

PCL-5	Baseline	20	55.26	1.97	59.27 to 58.16	19	54.16	1.97	50.16 to 58.16	1.10	-4.85 to 7.05	0.708
	8 weeks	12	22.63	5.53	11.08 to 34.17	16	41.87	4.65	32.17 to 51.575	19.245	3.30 to 35.19	**0.020**
	16 weeks	10	22.94	6.70	8.02to 37.86	7	27.77	8.72	8.34 to 47.20	4.83	-20.06 to 29.72	0.675

WSAS	Baseline	19	23.40	2.03	19.27 to 27.53	18	21.66	2.03	17.52 to 25.79	1.74	-4.42 to 7.90	0.568
	8 weeks	11	10.86	3.54	3.46 to 18.26	16	22.69	2.76	16.91 to 28.48	11.83	1.52 to 22.14	**0.027**
	16 weeks	9	11.87	3.61	3.55 to 20.19	6	20.84	5.09	9.10 to 32.57	8.97	-6.51 to 24.45	0.218

PHQ-9	Baseline	20	14.44	1.38	11.64 to 17.25	19	17.88	1.38	15.07 to 20.68	3.43	-0.65 to 7.52	0.097
	8 weeks	12	6.94	1.93	2.92 to 10.97	16	13.58	1.62	10.20 to 16.96	6.63	1.08to 12.19	**0.022**
	16 weeks	10	7.24	2.14	2.48 to 12.01	7	9.27	2.79	3.06 to 15.47	2.03	-5.93 to 9.98	0.583

GAD-7	Baseline	20	13.85	0.76	12.30 to 15.40	19	13.89	0.76	12.34 to 15.43	0.04	-2.26 to 2.34	0.974
	8 weeks	12	5.95	1.82	2.16 to 9.74	16	12.44	1.53	9.25 to 15.62	6.48	1.25 to 11.72	**0.018**
	16 weeks	10	6.88	1.87	2.71 to 11.04	7	6.37	2.43	0.95 to 11.79	0.51	-6.44 to 7.46	0.874

ISI	Baseline	20	18.27	1.36	15.49 to 21.05	19	18.26	1.36	15.48 to 21.03	0.01	-4.11 to 4.14	0.995
	8 weeks	12	10.37	2.52	5.12 to 15.62	16	15.93	2.12	11.52 to 20.34	5.56	-1.69 to 12.82	0.125
	16 weeks	10	10.43	2.53	4.79 to 16.07	7	13.79	3.30	6.44 to 21.13	3.36	-6.05 to 12.77	0.445

Audit-O	Baseline	18	5.16	1.95	1.17 to 9.15	17	5.02	1.95	1.03 to 9.01	0.14	-5.75 to 6.03	0.961
	8 weeks	11	3.30	1.62	-0.10 to 6.69	16	4.47	1.30	1.75 to 7.19	1.17	-3.37 to 5.71	0.596
	16 weeks	10	3.41	1.23	0.67 to 6.16	7	2.31	1.60	-1.26 to 5.89	1.10	-3.48 to 5.68	0.605

MSPSS	Baseline	20	55.59	3.88	47.68 to 63.50	19	55.68	3.88	47.77 to 63.59	0.09	-11.66 to 11.84	0.988
	8 weeks	12	60.59	5.34	49.41 to 71.77	15	52.82	4.67	43.04 to 62.60	7.77	-7.87 to 23.40	0.311
	16 weeks	10	66.03	4.87	55.19 to 76.87	7	47.79	6.34	33.67 to 61.91	18.24	0.15 to 36.32	**0.048**

Bold has been used to highlight results that were statistically significant at the *α* = 0.05 level. Model covariates: baseline version of outcome, gender, and baseline PHQ-9 (for comorbidity of depression) (removed for analysis of PHQ-9 as an outcome else duplicated from before) and time since trauma (in months).

## Data Availability

Data are available on request.

## Supplementary Materials

Supplementary Materials Table 1: CONSORT 2010 checklist of information to include when reporting a pilot or feasibility trial. Supplementary Table 2: CAPS-5 categorical diagnosis at multiple time points. Supplementary Table 3: attrition rates over time between the intervention and control groups.
