# Supplementary material for: Rewind for Posttraumatic Stress Disorder: A Randomised Controlled Trial
Source: Depress Anxiety. 2023 Oct 12;2023:6279649. doi: 10.1155/2023/6279649 (PMC11921860; doi:10.1155/2023/6279649)
Supplement: Supplementary Materials — Table 1: CONSORT 2010 checklist of information to include when reporting a pilot or feasibility trial. Supplementary Table 2: CAPS-5 categorical diagnosis at multiple time points. Supplementary Table 3: attrition rates over time between the intervention and control groups. [file 6279649.f1.docx]

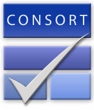
SUPPLEMENTARY TABLE 1: CONSORT 2010 checklist of information to include when reporting a pilot or feasibility trial*

| Section/Topic | Item No | Checklist item | Reported on page No |
| --- | --- | --- | --- |
| Title and abstract | | | |
|  | 1a | Identification as a pilot or feasibility randomised trial in the title | 1 |
|  | 1b | Structured summary of pilot trial design, methods, results, and conclusions (for specific guidance see CONSORT abstract extension for pilot trials) | 1 |
| Introduction | | | |
| Background and objectives | 2a | Scientific background and explanation of rationale for future definitive trial, and reasons for randomised pilot trial | 2 |
|  | 2b | Specific objectives or research questions for pilot trial | 2 |
| Methods | | | |
| Trial design | 3a | Description of pilot trial design (such as parallel, factorial) including allocation ratio | 2-3 |
|  | 3b | Important changes to methods after pilot trial commencement (such as eligibility criteria), with reasons | 2-3 |
| Participants | 4a | Eligibility criteria for participants | 2-3 |
|  | 4b | Settings and locations where the data were collected | 3 |
|  | 4c | How participants were identified and consented | 3 |
| Interventions | 5 | The interventions for each group with sufficient details to allow replication, including how and when they were actually administered | 3-4 |
| Outcomes | 6a | Completely defined prespecified assessments or measurements to address each pilot trial objective specified in 2b, including how and when they were assessed | 3 |
|  | 6b | Any changes to pilot trial assessments or measurements after the pilot trial commenced, with reasons | n/a |
|  | 6c | If applicable, prespecified criteria used to judge whether, or how, to proceed with future definitive trial | n/a |
| Sample size | 7a | Rationale for numbers in the pilot trial | 2 |
|  | 7b | When applicable, explanation of any interim analyses and stopping guidelines | n/a |
| Randomisation: |  |  |  |
| Sequence  generation | 8a | Method used to generate the random allocation sequence | 3 |
|  | 8b | Type of randomisation(s); details of any restriction (such as blocking and block size) | 3 |
| Allocation  concealment  mechanism | 9 | Mechanism used to implement the random allocation sequence (such as sequentially numbered containers), describing any steps taken to conceal the sequence until interventions were assigned | 3-4 |
| Implementation | 10 | Who generated the random allocation sequence, who enrolled participants, and who assigned participants to interventions | 3 |
| Blinding | 11a | If done, who was blinded after assignment to interventions (for example, participants, care providers, those assessing outcomes) and how | 3 |
|  | 11b | If relevant, description of the similarity of interventions | n/a |
| Statistical methods | 12 | Methods used to address each pilot trial objective whether qualitative or quantitative | 2-3 |
| Results | | | |
| Participant flow (a diagram is strongly recommended) | 13a | For each group, the numbers of participants who were approached and/or assessed for eligibility, randomly assigned, received intended treatment, and were assessed for each objective | 6 |
|  | 13b | For each group, losses and exclusions after randomisation, together with reasons | 6 |
| Recruitment | 14a | Dates defining the periods of recruitment and follow-up | 3 |
|  | 14b | Why the pilot trial ended or was stopped | n/a |
| Baseline data | 15 | A table showing baseline demographic and clinical characteristics for each group | 7 |
| Numbers analysed | 16 | For each objective, number of participants (denominator) included in each analysis. If relevant, these numbers  should be by randomised group | 6 |
| Outcomes and estimation | 17 | For each objective, results including expressions of uncertainty (such as 95% confidence interval) for any  estimates. If relevant, these results should be by randomised group | 9-11 |
| Ancillary analyses | 18 | Results of any other analyses performed that could be used to inform the future definitive trial | 9-11 |
| Harms | 19 | All important harms or unintended effects in each group (for specific guidance see CONSORT for harms) | 9 |
|  | 19a | If relevant, other important unintended consequences | 9 |
| Discussion | | | |
| Limitations | 20 | Pilot trial limitations, addressing sources of potential bias and remaining uncertainty about feasibility | 12-13 |
| Generalisability | 21 | Generalisability (applicability) of pilot trial methods and findings to future definitive trial and other studies | 13-14 |
| Interpretation | 22 | Interpretation consistent with pilot trial objectives and findings, balancing potential benefits and harms, and  considering other relevant evidence | 12-14 |
|  | 22a | Implications for progression from pilot to future definitive trial, including any proposed amendments | 14 |
| Other information | | |  |
| Registration | 23 | Registration number for pilot trial and name of trial registry | 3 |
| Protocol | 24 | Where the pilot trial protocol can be accessed, if available | 1 |
| Funding | 25 | Sources of funding and other support (such as supply of drugs), role of funders | 14-15 |
|  | 26 | Ethical approval or approval by research review committee, confirmed with reference number | 3 |

| **Supplementary Table 2: CAPS-5 categorical diagnosis at multiple time points** | | | | | |
| --- | --- | --- | --- | --- | --- |
|  | | **Immediate n (%)** | **Delayed n (%)** | **Total n (%)** | **Completers n** |
| **CAPS-5 Categorical Diagnosis Baseline** | **Positive** | 20 (100) | 20 (100) | 40 (100) | 40 |
|  | **Negative** | 0 (0) | 0 (0) | 0 (0) |  |
| **CAPS-5 Categorical Diagnosis 8 weeks** | **Positive** | 7 (35) | 12 (60) | 19 (47.5) | 32 |
|  | **Negative** | 10 (50) | 3 (15) | 13 (32.5) |  |
| **CAPS-5 Categorical Diagnosis 16 weeks** | **Positive** | 6 (30) | 2 (10) | 8 (20) | 26 |
|  | **Negative** | 11 (55) | 7 (35) | 18 (45) |  |

| **Supplementary Table 3: Attrition rates over time between intervention and control groups** | | |
| --- | --- | --- |
|  | **Intervention n = 20** | **Control n = 20** |
| **Attended 1 session of Rewind technique n (%)** | 20 (100) | 18 (90) |
| **Attended 2 sessions of Rewind technique n (%)** | 19 (95) | 12 (60) |
| **Attended 3 sessions of Rewind technique n (%)** | 17 (85) | 7 (35) |
| **Completed CAPS-5 baseline n (%)** | 20 (100) | 20 (100) |
| **Completed CAPS-5 8 week n (%)** | 17 (85) | 15 (75) |
| **Completed CAPS-5 16 week n (%)** | 17 (85) | 9 (45) |
| **Completed secondary measures via online survey baseline n (%)** | 20 (100) | 19 (95) |
| **Completed secondary measures via online survey 8 week n (%)** | 12 (60) | 16 (80) |
| **Completed secondary measures via online survey 16 week n (%)** | 10 (50) | 7 (35) |
